# Transcytosis to Cross the Blood Brain Barrier, New Advancements and Challenges

**DOI:** 10.3389/fnins.2018.01019

**Published:** 2019-01-11

**Authors:** Victor M. Pulgar

**Affiliations:** ^1^Department of Pharmaceutical Sciences, Campbell University, Buies Creek, NC, United States; ^2^Department of Obstetrics and Gynecology, Wake Forest School of Medicine, Winston-Salem, NC, United States

**Keywords:** brain endothelium, transcellular, receptor-mediated transcytosis, drug delivery, CNS diseases

## Abstract

The blood brain barrier (BBB) presents a formidable challenge to the delivery of drugs into the brain. Several strategies aim to overcome this obstacle and promote efficient and specific crossing through BBB of therapeutically relevant agents. One of those strategies uses the physiological process of receptor-mediated transcytosis (RMT) to transport cargo through the brain endothelial cells toward brain parenchyma. Recent developments in our understanding of intracellular trafficking and receptor binding as well as in protein engineering and nanotechnology have potentiated the opportunities for treatment of CNS diseases using RMT. In this mini-review, the current understanding of BBB structure is discussed, and recent findings exemplifying critical advances in RMT-mediated brain drug delivery are briefly presented.

## Introduction

Brain diseases are among the less understood and poorly treated conditions. In spite of the rapid growth in recent years in drug development, there is still a low success rate of effective therapies focused in diseases of the central nervous system. A main issue hindering therapeutic success is the tightly regulated extracellular environment of the brain tissue which makes reaching macromolecular targets into the brain a great challenge (Pardridge, 2005; Abbott, 2013; Engelhardt et al., 2016). The isolation of the brain tissue from the peripheral circulation is thought to arise from the existence of multi-level “barriers,” established in different compartments in the central nervous system of most vertebrates (Cserr and Bundgaard, 1984; Engelhardt et al., 2017) providing protection to the neural tissue. Key to those protective mechanisms is the regulation of the entry of macromolecules from the blood to the brain across the blood-brain barrier (BBB) (Abbott et al., 2006). The BBB regulates an extended surface of interaction between blood and brain. It is calculated that the brain capillary network in humans is approximately 600 km long with a surface of 15–25 m^2^ (Wong et al., 2013).

The intimate association between neurons, glial cells, and brain microvessels in the neurovascular unit is being recognized as the functional point for regulation of cerebral blood flow. Among those cell types, the brain endothelial cells are the building blocks of the BBB impeding the entry of most molecules from blood to brain, with the exception of those small and lipophilic in nature. Several recent studies have focused on the functional interactions between endothelial, neuronal and glial cell types and their role on regulating BBB function (Persidsky et al., 2006; Chow and Gu, 2015; Liebner et al., 2018). Since neurons rarely occur at long distance from a brain capillary (Schlageter et al., 1999; Tsai et al., 2009), the BBB also plays a major role in controlling fast delivery of substances to the brain and the local neuronal environment. Due to its extended contact and exchange surface area, most research has focused on the brain endothelium as the therapeutic target to increase brain drug delivery.

## Structure of the Bbb

Structurally, capillary networks can be divided into continuous non-fenestrated capillaries, continuous fenestrated and discontinuous capillaries. This division is based on their ability to regulate crossing of solutes from blood to tissues; thus continuous fenestrated capillaries are least permeable whereas discontinuous are the most permeable (Aird, 2007a,b). In the BBB, continuous non-fenestrated capillaries, where tight junctions (TJs) connect endothelial cells, form a high-resistance para-cellular barrier limiting the crossing of molecules and ions. Transmembrane proteins are an important part of TJs, they bind the cytoskeleton and link adjacent endothelial cells in a close configuration, eliminating intercellular spaces. Some of the proteins important for TJs structure and function include integral membrane proteins such as members of the claudin family i.e., claudin 3, 5, and 12, ocludins, and junctional adhesion molecules (Anderson and Van Itallie, 2009; Furuse, 2010). Evidences indicate that claudins are essential for the formation of the para-cellular barrier and the structure is stabilized by zona occludens ZO -1, -2, and -3 and additional proteins that link the TJs with the cytoskeleton (Abbott et al., 2006; Furuse, 2010). This structure is further reinforced by the basal lamina, a ∼40 nm thick matrix formed predominantly of collagen type 1V, laminin, and heparan sulfate proteoglycan (Perlmutter and Chui, 1990). Metalloproteinases are other components that contribute to regulation of BBB function in health and disease (Yong, 2005).

Additionally, glial cells such as astrocytes play an important role in development and maintenance of the BBB. Up to 99% of the basal capillary membrane is covered by astrocytes “end feet” and glial-derived factors such as GDNF, angiopoietin-1 and angiotensin II all contribute to BBB integrity (Hori et al., 2004; Abbott et al., 2006; Wosik et al., 2007). Along with astrocytes “end feet,” pericytes are also lining the cerebral vasculature, surrounding brain endothelial cells and contributing to the barrier properties of the BBB. Recent advances on pericytes research indicate that this cell type is rather complex with more than one functional definition depending on their location along the arterio-venous capillaries (Attwell et al., 2016). The fact that brain microvessels are enriched in pericytes, and pericyte-deficient mouse mutants showed increased BBB permeability (Armulik et al., 2010) exemplifies the importance of pericytes for BBB control. Pericytes seem to contribute in two ways to BBB integrity: downregulating *trans*-endothelial permeability and promoting astrocyte-endothelial cells contacts (Armulik et al., 2010). Moreover, growing evidences point now to the importance of the interactions between pericytes and other cell types within the neurovascular unit in health and disease (ElAli et al., 2014).

The multicellular organization occurring at the neurovascular unit involving endothelial cells and astrocytes among others cell types (Willis, 2012) forms the framework where the highly regulated crossing of macromolecules from blood to brain occurs.

## Crossing the Bbb

**FIGURE 1:**
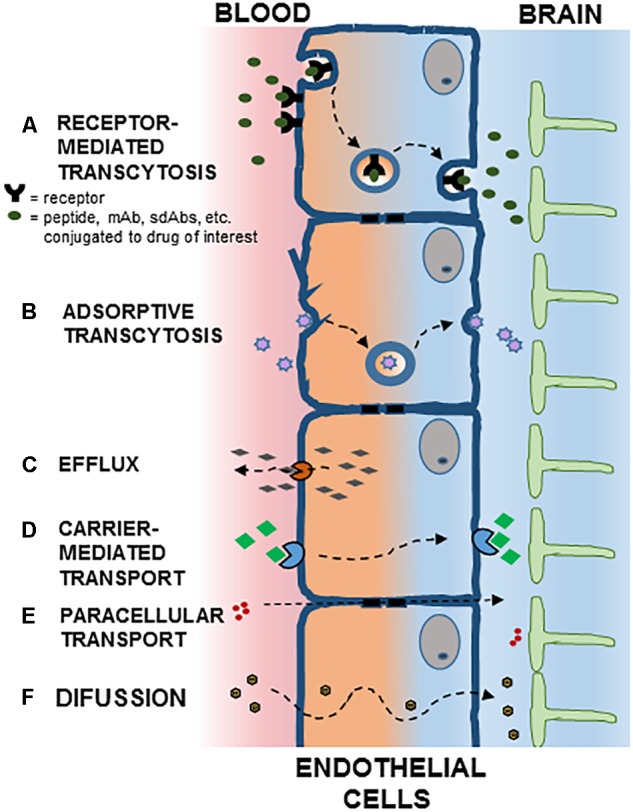
Potential mechanisms for crossing the blood brain barrier (BBB). Polarized endothelial cells, bound by tight junctions, form a seal that controls free movement or molecules from blood to brain. In brain capillaries, endothelial cells are in intimate association with astrocytes. Potential mechanisms for crossing the BBB are indicated: **(A)** Receptor Mediated Transcytosis; **(B)** Adsorptive Transcytosis; **(C)** Efflux; **(D)** Carrier-Mediated Transport; **(E)** Paracellular Transport; **(F)** Diffusion. See text for details.

The existence of efflux transport systems in brain capillary endothelial cells reinforce the barrier properties of the BBB by removing undesirable substances from the brain to the systemic circulation. Multidrug resistance transporters, monocarboxylate transporters and organic anion transporters/organic anion transporting polypeptide have been implicated in the efflux of drugs from the brain. Consequently, the activity of these efflux transporters limits the effectiveness of CNS targeted drugs (Loscher and Potschka, 2005; Figure 1C).

Most of the drug transporters belong to two major classes; adenosine triphosphate binding cassette (ABC) and solute carrier (SLC) transporters. ABC transporters are active transporters coupling efflux against concentration gradients to ATP hydrolysis with P-glycoprotein (P-gp) being the most extensively studied BBB transporter of the ABC family (Mahringer and Fricker, 2016). P-gp is encoded by the multidrug resistance gene 1 (MDR1) and its function is regulated by intracellular factors and environmental toxins (Dauchy et al., 2009).

In order to facilitate the efficient delivery of drugs to the brain, the functional and structural tightness of the BBB needs to be overcome. Strategies used to cross BBB involve para-cellular as well as *trans*-cellular mechanisms.

## Transport Across the Bbb

As part of its normal function, the endothelial cells allow the influx of nutrients and regulatory molecules into the brain via passive and active mechanisms. In normal conditions, some passive movement of solutes exists through small intercellular pores located in the TJs (Figure 1E). The molecular entities responsible for this transport are largely unknown, although recent evidences point to claudins as pore-forming structures in BBB TJs (Irudayanathan et al., 2017). Since early stage CNS diseases do not show evident BBB alterations, this pathway offers fewer opportunities than *trans*-cellular transport for drug delivery.

Transport of small molecules trough cells is common in polarized cells. Thus, in brain vascular endothelial cells, hydrophobic molecules with molecular weight lower than 500 Da once they escape the P-gp-type multidrug resistance efflux pumps may diffuse transcellularly from systemic circulation to brain parenchyma (Figure 1F). The transport of nutrients, however, requires specialized transporters (Figure 1D). Thus, large neutral aminoacid transporters (LAT1) transport aminoacids, nucleosides, and some drugs, while glucose uses the glucose transporter (GLUT1) (Ohtsuki and Terasaki, 2007; Barar et al., 2016).

## Transcytosis

Transcytosis is a phenomenon present in many different cell types, from neurons to intestinal cells, osteoclasts and endothelial cells. In polarized cells, unidirectional transcytosis refers to the transport of macromolecules from apical to basolateral plasma membranes. Steps along this pathway include endocytosis, intracellular vesicular trafficking and exocytosis. The first of these steps may involve adsorptive (charge dependent) or receptor-mediated internalization (Figures 1A,B). Positively charged molecules such as polymers, cationic lipids, albumin and nanoparticles may interact with the negatively charged cell membrane and internalize through adsorptive endocytosis (Lu, 2012). Although initially thought to be attenuated in brain endothelial cells, virtually all endothelial cells display receptor-mediated transcytosis (RMT) (Stewart, 2000). Recent applications of imaging techniques allowed for detailed analyses of transcytosis in brain endothelial cells (Villasenor and Collin, 2017). Several receptors capable of inducing RMT are present in the BBB, such as the insulin receptor, transferrin receptor, and receptors responsible for lipoprotein transport, while others such as albumin receptors are not expressed (Pardridge et al., 1985).

The intracellular transport of macromolecules is mediated by the vesicular system (Parkar et al., 2009). In brain endothelial cells three types of endocytic vesicles have been identified: *clathrin-coated pits* involved in most of the RMT, *caveolae* that participate in adsorptive-mediated endocytosis of extracellular molecules and receptor trafficking, and *macropinocytotic vesicles* (Mayor and Pagano, 2007). Of these, clathrin-coated vesicles are involved in most of the internalization processes mediated by approximately 20 different receptors in brain endothelial cells.

**FIGURE 2:**
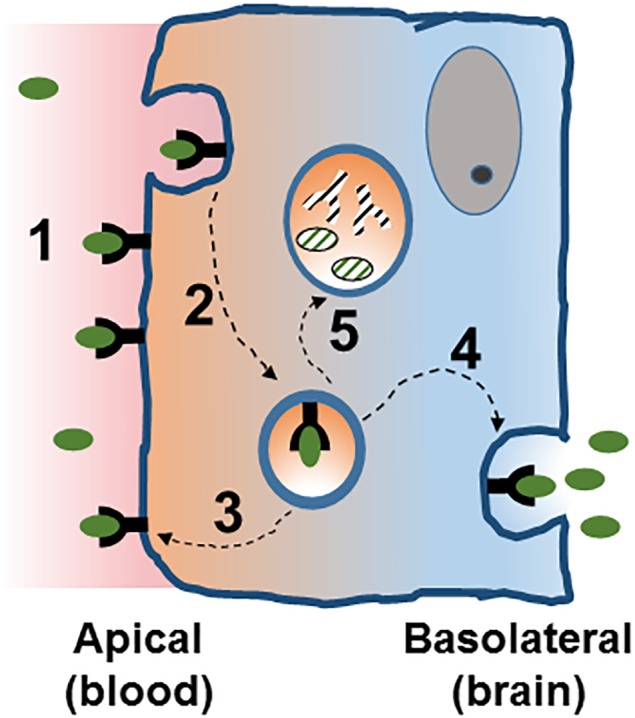
Receptor mediated transcytosis in the BBB. A ligand binds its cognate receptor at the apical membrane of the brain endothelial cell (1), and initiates the invagination of the plasma membrane and the endocytosis process (2). Intracellularly, the vesicle can follow different traffic routes including recycling to the apical membrane (3) or routing to the basolateral membrane where membrane fusion allows for the release of the vesicle content [transcytosis, (4)]. Routing of the vesicle to the lysosome (5) would target it for degradation. See text for details.

Once a vesicle is internalized, the common intracellular pathway begins with the initial sorting station, the early endosome (Rodriguez-Boulan et al., 2005; Brooks, 2009; Figure 2). In BBB endothelial cells endocytosis occurs at the apical and basolateral membranes with both processes generating its own early endosomes. In polarized cells, routing back to the plasma membrane can occur directly from EE or from recycling endosomes (Thompson et al., 2007). Alternatively, vesicle components can be delivered to late endosomes and targeted for lysosomal degradation. This endosomal trafficking plays an important role in the efficiency of RMT in BBB (Haqqani et al., 2018).

## Rmt for Drug Delivery to the Brain

In general, strategies using RMT for drug delivery to the brain involve the generation of a complex between the drug of interest and a receptor-targeting entity. This entity may be the endogenous receptor ligand, an antibody targeting the receptor or a mimetic peptide ligand. These two components can be chemically linked or the drug can be incorporated in liposomes or nanoparticles decorated with the RMT-targeting ligand (Jones and Shusta, 2007). Among the most studied targets for RMT in brain endothelial cells are the transferrin receptor, low-density lipoprotein (LDL) receptor and insulin receptor, for reviews see (Lajoie and Shusta, 2015; Paterson and Webster, 2016). In the following section, some examples of the use of these systems are presented with focus in recent advances.

### Transferrin Receptor

Iron delivery to the brain is accomplished via binding and intracellular trafficking of the iron binding protein transferrin (Tf). The Tf receptor (TfR) has been the target of numerous *in vitro* and *in vivo* studies aiming to deliver drugs to the brain (see Table 1). Approaches used include liposomes decorated with Tf used for delivery of imaging agents and DNA (Sharma et al., 2013) or the use of an iron-mimetic peptide as ligand (Staquicini et al., 2011). Since the presence of high blood levels of Tf requires competition with the endogenous ligand, alternative methods involving anti-TfR antibodies have been developed (Qian et al., 2002). Challenges using anti-TfR to deliver drugs to the brain via RMT include specificity to the brain tissue, potential lysosomal degradation and significant transport into the brain parenchyma. With the use of protein engineering it has been shown that reducing antibody’s affinity for Tf improves release of the antigen-antibody complex in the basolateral side of the BBB endothelial cells (Yu et al., 2011). A correlation has also been suggested between increased antibody’s affinity and lysosomal degradation (Bien-Ly et al., 2014) supporting the idea that lower antibody’s affinity would help avoid intracellular degradation of the complexes being transported. Studies comparing the brain penetration of monovalent versus divalent antibodies indicate lower lysosomal colocalization of the monovalent form (Niewoehner et al., 2014) and better transcytosis (Johnsen et al., 2018). It appears that in addition to antibody’s affinity in physiological conditions, a lower affinity at pH5.5 (lysosomal) also promotes effective transcytosis as suggested by *in vitro* studies using immortalized human brain endothelial cells (Sade et al., 2014).

**Table 1:** Main receptor systems identified mediating receptor-mediated transcytosis (RMT) cargo delivery through the BBB.

Receptor targeted in RMT	Biological effect	Reference
**Transferrin Receptor (TfR)**		
Cyclic iron-mimicking peptide as RMT ligand (CRTIGPSVC).	HSV-Thymidine kinase gene specifically delivered to mouse brain tumors through a non-canonical allosteric binding mechanism to TfR.	Staquicini et al., 2011
Liposomes decorated with Tf-poly-L-arginine loaded with imaging agents or β-gal expressing plasmid.	4% of injected dose of imaging agents reached the brain 24 h after i.v., injection. Greater β-gal compared to injection of naked DNA.	Sharma et al., 2013
PEGylated liposomes decorated with anti-TfR antibody (8D3) loaded with plasmid encoding *β*-glucoronidase.	At 48 h post i.v., injection tenfold higher *β*-glucoronidase activity observed in brain, liver and spleen in mouse model of mucopolysaccharidosis VII.	Zhang et al., 2008
cTfRMAb (chimeric anti-mouse TfR monoclonal antibody) complexed with tumor necrosis factor receptor (TNFR): cTfRMAb-TNFR.	Mice model of Parkinson’s disease (PD) i.v., treated for 3 weeks showed 130% increase in striatal tyrosine hydroxylase (TH) and improvements in behavioral testing.	Zhou et al., 2011a
PEGylated chitosan nanoparticles decorated with anti-TfRMAb (R17-217): CS-PEG-BIO/SA-TfRMAb.	Decreased infarct volume, neurological deficit, and ischemia-induced caspase-3 activity in mice model of stroke i.v., injected with CS-PEG-BIO/SA-TfRMAb.	Karatas et al., 2009
cTfRMAb complexed with erythropoietin (EPO): cTfRMAB-EPO. cTfRMAb complexed with glial-derived neurotrophic factor (GDNF): cTfRMAB-GDNF.	Mouse model of PD i.v., treated for 3 weeks showed >300% and >250% increase in striatal TH, respectively and improvements in behavioral testing.	Fu et al., 2010; Zhou et al., 2011b
Liposomes decorated with anti-TfR loaded with GDNF-expressing plasmids.	Rat model of PD i.v., treated showed 77% increase in TH activity and neurobehavioral improvements.	Zhang and Pardridge, 2009
cTfRMAb complexed with single chain Fv (ScFv) antibody: cTfRMAb-ScFv.	Bi-functional binding to TfR and Aβ, accumulation in mouse brain >3%ID/g. Mouse model of Alzheimer’s disease (AD) showed 40–60% reduction in Aβ fibrils.	Boado et al., 2010; Sumbria et al., 2013
Monovalent binding anti-TfR antibody. “Brain Shuttle” antibody for AD.	Enhanced RMT compared to bivalent Ab. Increased destruction of β-Amyloid plaques in mouse model of AD. Changes in binding mode attenuated peripheral effects.	Niewoehner et al., 2014; Weber et al., 2018
High (anti-TfR^A^/BACE1) and low (anti-TfR^D^/BACE1) affinity bispecific antibodies anti TfR and β-amyloid cleaving enzyme-1 (BACE1).	In WT mice i.v., injected, high-affinity binding to TfR caused a dose-dependent reduction of brain TfR levels and lysosomal degradation of TfR.	Bien-Ly et al., 2014
Variants of the 8D3 anti-TfR with reduced affinity fused with IL-1 receptor antagonist IL-1RA: IgG1TM-IL-1RA.	Male C57B/l mice i.v., injected with IgG1TM-IL-1RA showed 22 to 69-fold greater brain content of lower affinity variants vs. 8D3. Reverse of mechanical hyperalgesia also observed.	Webster et al., 2017
Human TfR fused to iduronate 2-sulfatase (IDS): JR-141.	Immunoreactivity of JR-141 found in brain in *TFRC*-KI/*Ids*-KO mice. Phase I/II clinical trial of JR-141 for mucopolysaccharidosis II (MPSII) currently underway.	Sonoda et al., 2018
**Insulin Receptor (IR)**		
HIRMAb fused to a single chain anti Amyloid β antibody (scFv): HIRMAb-scFv.	Transport to the brain in Rhesus monkeys with a brain uptake of approximately 1% injected dose (ID)/100 g tissue.	Boado et al., 2010
HIRMAb fused to GDNF: HIRMAb-GDNF.	In parkinsonian monkeys twice a week 3-mo i.v., injections of HIRMAb-GDNF did not improve parkinsonian motor symptoms and induced a dose-dependent hypersensitivity reaction.	Ohshima-Hosoyama et al., 2012
HIRMAb fused to iduronate 2-sulfatase (IDS): HIRMAb-IDS.	Brain uptake in Rhesus monkeys approximately 3% ID/100 g tissue. No toxicity observed during a 6-month treatment study.	Lu et al., 2011; Boado et al., 2014a
HIRMAb fused to paraoxonase (PON)-1: HIRMAb-PON1.	Fusion protein detected in brain in Rhesus monkeys after i.v., injection.	Boado et al., 2011
HIRMAb complexed with arylsulfatase (ASA): HIRMAb-ASA.	In Rhesus monkeys i.v., injected, brain uptake of 1.1 and 0.32% ID/100 g in gray and white matter, respectively., HIRMAb-ASA observed in all parts of brain.	Boado et al., 2013
HIRMAb complexed with *N*-sulfoglucosamine sulfohydrolase (SGSH): HIRMAb-SGSH.	72–83% reduction in lysosomal glycoso-aminoglycans in mucopolysaccharidosis type III (MPSIIIA) fibroblasts. In Rhesus monkeys i.v., injected, brain uptake of ∼1% ID/100 g. Reduction in brain heparan sulfate in MPSIIIA mouse.	Boado et al., 2014b, 2018
Human anti-IR antibody (HIRMAb) complexed with iduronidase: HIRMAb-IDUA, Valanafusp, AGT-181.	In a Phase II trial, 11 children with mucopolysaccharidosis type I (MPSI), a lysosomal storage disease, showed evidences of cognitive and somatic stabilization.	Giugliani et al., 2018
**Low Density Lipoprotein Receptor (LDLR)**		
Nanoparticles decorated with apolipoprotein A (ApoE).	ApoE-modified nanoparticles cross BBB in brain capillary endothelial cells.	Wagner et al., 2012
Lentivirus vector encoding amyloid β- degrading enzyme neprilysin fused to ApoB transport domain.	Mouse model of AD showed reduced Aβ and plaques levels.	Spencer et al., 2011
Sulphamidase fused to secretion signal peptide of iduronate-2-sulphatase (IDS) and ApoB-binding domain.	Single i.v., injection on MPSIIIA mice showed efficient BBB transcytosis and restoration of sulphamidase activity in the brain.	Sorrentino et al., 2013
Lentiviral IDS fused to ApoEII (IDS.ApoEII) used in stem cell therapy.	MPSII mice showed normalization of brain pathology and behavior, including correction of astrogliosis and lysosomal swelling.	Gleitz et al., 2018
Family of Kunitz domain-derived peptides with BBB crossing capacity.	Angiopep-2 peptide cross the BBB by interaction with LPR1, reaching brain parenchyma.	Demeule et al., 2008
Angiopep-2 combined with antitumor drug paclitaxel: (ANG1005, GRN1005).	Phase I study in recurrent malignant glioma patients showed brain delivery of drug with therapeutic activity.	Drappatz et al., 2013
ANG1005 in brain metastases of breast cancer.	Rat models of breast cancer showed improved brain uptake through BBB transcytosis.	Thomas et al., 2009
ANG1005 in brain metastases of breast cancer.	Imaging study of ANG1005 in human patients to treat breast cancer metastasis to the brain	O’Sullivan et al., 2016
**Single domain llama antibodies (FC5, FC44)**		
Single domain FC5 antibody.	BBB transcytosis of FC5 is dependent on clathrin-coated endocytic vesicles and on the recognition α(2,3)-sialoglycoprotein receptor on human endothelial cells.	Abulrob et al., 2005
Single domain FC5 antibody.	MS based methods showed that systemic administration in rats produces highly facilitated BBB transport of FC5.	Haqqani et al., 2013
Bispecific antibody FC5-mGluR1 (BBB-mGluR1).	After i.v., injection in rats >tenfold higher accumulation of BBB-mGluR1 in brain, and suppression of thermal hyperalgesia.	Webster et al., 2016


*Evidences of efficient RMT utilizing the Transferrin Receptor, Insulin Receptor, Low Density Lipoprotein Receptor, and single domain llama antibodies are summarized.*

The recent successes using TfR in RMT strategies has prompted novel developments aiming to potentiate drug delivery to the brain (Yemisci et al., 2018). Thus, recent reports showed efficient BBB crossing of particles functionalized with anti-TfR antibodies and containing non-permeant drugs of interest for treating brain diseases. Some examples include liposomes containing the MYBE/4C1 antihuman TfR antibody and loaded with the anticancer drug doxorubicin displaying enhanced uptake in human brain endothelial cells (Gregori et al., 2016), and liposomes containing Tf and docetaxel showing greater brain uptake after i.v., injection in rats compared to the drug alone (Sonali et al., 2016). The use of nanoparticles formulated using the Tf system has shown that functionalization with anti-TfR antibodies enhances the delivery of particles carrying relevant drugs such as drugs able to inhibit beta amyloid aggregates (Loureiro et al., 2016). Nanoparticles carrying the chemotherapeutic agent temozolomide have also facilitated enhanced drug uptake by glioblastoma cells (Ramalho et al., 2018). This strategy also exemplifies some of the challenges remaining in the field since gold nanoparticles (AuNPs) coated with the 8D3 anti-TfR antibody injected in mouse are transported through the BBB with low efficiency and most of the particles remain sequestered intracellularly in the endothelial cells (Cabezon et al., 2015). Successful uptake by the BBB but low delivery to the brain parenchyma was also reported with quantum dots (Paris-Robidas et al., 2016). The dual functionalization of particles with peptides targeting the TfR to cross the BBB and additional therapeutic agents opens opportunities to specifically modulate gene expression in brain cells as shown by studies of co-delivery of doxorubicin and RNAi targeting the VEGF (Kuang et al., 2016), or siRNA targeting the EGFR (Wei et al., 2016) to glioma cells. The significant reduction in expression of the pro vascularization factors VEGF and EGFR observed in these two studies supports this use of co-delivery systems.

TfR has been used extensively as a model for brain transcytosis, although initial reports came from just one laboratory, later reports supported reproducibility of its use in different settings. Outstanding issues remaining such as brain specificity and low drug uptake will promote further research of this important RMT system.

### Insulin Receptor

Insulin is transported into the brain by the insulin receptor (IR). Similarly to the TfR, anti-IR antibodies have been developed and used in strategies to drug delivery into the brain (see Table 1). Following the development of humanized anti-IR antibodies (HIRMAb) that showed good internalization and transport to the brain after intravenous administration in monkeys (Boado et al., 2007), fusion proteins were developed to deliver relevant enzymes as therapies for genetic disorders. One of those examples is a fusion protein between the HIRMAb and α-L-idorunidase (IDUA) an enzyme missing in Hurler’s Syndrome, Mucopolysaccharidosis Type I (MSPI), a disorder of brain lysosomal storage (Boado et al., 2008). In pre-clinical studies, HIRMAb-IDUA showed good safety, adequate plasma glucose control, and limited antidrug antibody production (Boado et al., 2009, 2012). Of great interest are recent reports describing clinical studies with HIRMAb-IDUA. In MSPI pediatric and adult patients intravenous infusion of HIRMAb-IDUA describes the first clinical use of RMT to drug delivery into the brain (Pardridge et al., 2018). Although some adverse events reported include reaction at the infusion site, and transient hypoglycemia, the positive neurocognitive and somatic effects observed in pediatric patients (Giugliani et al., 2018) represents a significant advancement on the translational aspects of RMT.

### LDL Receptor

Low-density lipoprotein receptor (LDLR), a single transmembrane glycoprotein able to recognize LDL particles and promote their endocytosis, as well as LDLR-related proteins (LRPs), are present in the BBB and mediate transport of lipoproteins and other ligands through RMT (Hussain et al., 1999; Candela et al., 2008; Table 1). Recent *in vitro* studies showed that LDLR is preferentially located in apical rather than basolateral membranes in brain endothelial cells (Molino et al., 2017) supporting a role for ligand uptake from the circulation. To date no antibodies have been developed targeting the LDLR system, however, LDLR and LRP ligands have been used for drug delivery into the brain. One of those ligands is melanotransferrin, which displays a greater rate of brain transport compared to Tf. In spite of structural homology to Tf, melanotransferrin uses the LDLR and not the TfR to cross the BBB (Demeule et al., 2002). Interestingly, recent reports showed melanotrasferrin delivery and *in vivo* effectiveness of a fusion protein with an interleukin-1 receptor antagonist in a model of neuropathic pain (Thom et al., 2018). Lipoproteins have also been used to target LDLR for effective brain delivery (Wagner et al., 2012), as described in glioblastoma cells (Nikanjam et al., 2007). Recent developments include functionalization of solid nanoparticles with ApoE, these 160 nm nanoparticles showed efficient clathrin-dependent endocytosis and transcellular transport in human brain endothelial cells (Neves et al., 2017). Other targeting members of the LDLR family include “angiopeps.” For example, Angiopep-2 was identified by studying a series of 19 amino acid peptides with the ability to bind the LPR-1 receptor (Demeule et al., 2008). Angiopep-2 was shown to mediate efficient delivery of a conjugate Angiopep-2-placlitaxel to gliomas (Thomas et al., 2009), and more recently antinociceptive properties were demonstrated for an Angiopep-2-neurotensin fusion protein (Demeule et al., 2014). These studies provide evidence of successful delivery of therapeutically relevant agents to the brain via RMT targeting the LDLR family.

### Single Domain Llama Antibodies

Single domain antibodies (sdAbs) are naturally occurring fragments of the antibody’s heavy chain that lack the light chain. Among them, sdAbs from camelids specifically FC5 and FC44 have been studied for brain transcytosis of cargo in animal models and their potential warrants further developments (see Table 1). FC5 and FC44 recognize α(2,3)-sialoglycoprotein expressed in the luminal side of brain endothelial cells and display advantages over other antibodies such as small size, greater specificity and stability, and low immunogenicity (Arbabi-Ghahroudi, 2017).

## Conclusion

Recent advances using RMT are providing alternatives to overcome the barrier properties of the BBB and develop more efficient drug delivery to the brain. Future developments based the TfR, IR, and LDLR and other RMT systems will offer new opportunities in this growing field. However, in spite of clear therapeutic advances shown in animal studies, outstanding challenges remain for the development of efficient and specific RMT-based drug delivery. Although the mechanisms mediating efficient transcytosis through the brain endothelium are still incompletely understood, details about the specific targeting to brain endothelial cells are being revealed. Similarly, the limited brain specific versus systemic drug uptake may explain the lack of success of some potential therapies in non-human primates models of brain diseases.

In addition to increasing knowledge about the factors modulating intracellular trafficking, the generation of fusion proteins with RMT-targeting antibodies as well as functionalization of Nano carriers, an improved understanding of BBB transport, pharmacokinetics, and protein engineering will be needed to potentiate the clinical applicability of RMT.

## Author Contributions

VP was responsible for the design of this review, the literature searches, writing and interpretations presented.

## Conflict of Interest Statement

The author declares that the research was conducted in the absence of any commercial or financial relationships that could be construed as a potential conflict of interest.
